# Miscellany

**Published:** 1901-11

**Authors:** 


### Miscellany.

Local Anæsthesia without Risk of Danger.—Rasely (*International Journal of Surgery,* April, 1901) has found chloretone equally as good as cocaine in operative cases, including the incision of abscesses, amputation of fingers, and even in the extraction of teeth, without the least evidence of a depressing effect.

He has operated on a case of complete fistula in a man seventy-four years of age, with fatty degeneration of the heart, using chloretone as the anæsthetic for several reasons. No pain was felt, and there was not the slightest manifestation of depression or exhilaration, although syringeful after syringeful of the saturated aqueous solution was used in the line of the proposed incision.

Rasely thinks chloretone an ideal local anæsthetic, and he believes it to be sure in effect and safe in result. He thinks it is well adapted to take the place of cocaine in subarachnoid anæsthesia.—*Therapeutic Gazette.*

Curing a Boil.—A writer in the *Peoria Medical Journal* describes his method of treating boils as follows: "To render the process painless, a few drops of cocaine are introduced into the tissues and cavity of the boil, using a very fine needle, which is at first allowed to rest with its point upon the inflamed surface with merely the weight of the syringe to gradually force it beneath the skin. If there is a drop of cocaine upon the point of the needle, it will soon prepare the way for the further and more forcible introduction without pain, and then the cavity is made to receive enough to completely anæsthetize its surrounding tissue. When that is accomplished, a little larger needle is readily introduced and several drops of ninety-five per cent. carbolic acid are pressed tightly into the cavity. Absolute sterilization is invariably secured, and with absolutely no suffering to the patient whatever. A felon may be treated in the same way."—*Southern Medical Journal.*

---

To wax together a Broken Vulcanite Plate.—A dentist often needs three hands; for instance, when he essays to wax together the separate parts of a broken vulcanite plate. Two hands are necessary to properly hold the denture. My third hand, or assistant, is in the form of a device which holds the wax over the denture until it melts, and then lets it drop.

Take a bar of metal about one-twelfth of an inch thick, and solder to one end a small spoon-shaped piece large enough to hold as much wax as is ordinarily employed in waxing together the broken parts of the plate. While the other end of the bar is stuck into a hole in the table, or is held by a vice, let this spoon-shaped end be arched forward so as to rest about three inches above the table. Make a whole in the bottom of the spoon about one-twelfth of an inch in diameter. Into this little spoon place the wax. Then set an alcohol flame under the bar at an inch or two from the wax, so that the heat will creep down the bar to the wax. Soon it will melt and drop. The broken plate can thus be held steadily by two hands resting on the table.—Stewart J. Spence, *Dental Brief.*

---

A Method of securing Dam Clamps on Conically Shaped Teeth.—The adjusting of the rubber dam on some teeth, especially on some molars, is a vexatious undertaking. A method found to work nicely in the class called "conical" is as follows:

In order to overcome the difficulty and still use an ordinary clamp, have a varnish of shellac and alcohol. Place either a napkin or some of the absorbent materials in the mouth and around the tooth, and dry it as thoroughly as possible. Then apply some of the shellac varnish to both the buccal and lingual surfaces of the tooth, and allow it to dry. Saturate two pieces of spunk about twice the size of a pin-head in some chloropercha. Place one of these on the buccal and the other on the lingual surface of the tooth in a position to hold the clamp. In a few minutes the chloroform will evaporate and cause the spunk to adhere. Then the clamp and dam can be passed over the tooth and the two little spuds of spunk to position without the operator's being annoyed by the clamp working off the tooth. This is a simple but a very effectual remedy.—C. E. Bellchamber, D.M.D., *Dental Brief.*

[I have often in these cases employed sandarac varnish on the tooth for the purpose of securing a surface over which the clamp will not slip, to my great satisfaction.—McClain.]

